# AI for atmosphere–ocean sciences: advancements, challenges and ways forward

**DOI:** 10.1093/nsr/nwag063

**Published:** 2026-01-29

**Authors:** Jing-Jia Luo, Jiangjiang Xia, Baoxiang Pan, Yoo-Geun Ham, Xiaofeng Li, Wei Shangguan, Wei Xue, Yaqiang Wang, Bin Mu, Youngjoon Hong, Hao Li, Xiaohui Zhong, Kan Dai, Lei Bai, Fenghua Ling, Niklas Boers, Christopher Bretherton, Bin Chen, Dongjin Cho, Pierre Gentine, Zijie Guo, Xiaomeng Huang, Daehyun Kang, Hyunwoo J Kim, Jeong-Hwan Kim, Lili Lei, Fan Meng, Seol-Hee Oh, Bo Qin, Zixiong Shen, Qiming Sun, Yuheng Tang, Xuan Tong, Bingcheng Wan, Lina Wang, Ya Wang, Yiming Wang, Jiye Wu, Yi Xiao, Lina Yao, Song Yang, Chaoxia Yuan, Shijin Yuan, Tingzhao Yu, Mengchu Zhao

**Affiliations:** State Key Laboratory of Climate System Prediction and Risk Management (CPRM)/Jiangsu Key Laboratory of Intelligent Weather Forecasting and Applications Based on Big Data/ICAR/CIC-FEMD/KLME/ILCEC, School of Artificial Intelligence, Nanjing University of Information Science and Technology, Nanjing 210044, China; Temperate East Asia Research Center on Global Change, Institute of Atmospheric Physics, Chinese Academy of Sciences, Beijing 100029, China; National Key Laboratory of Earth System Numerical Modeling and Application, Institute of Atmospheric Physics, Chinese Academy of Science, Beijing 100029, China; Department of Environmental Management, Seoul National University, Seoul 08826, Republic of Korea; Institute of Oceanology, Chinese Academy of Sciences, Qingdao 266071, China; School of Atmospheric Sciences, Sun Yat-sen University, Guangzhou 510275, China; Department of Computer Science and Technology, Tsinghua University, Beijing 100084, China; Qinghai University and Intelligent Computing and Application Laboratory of Qinghai Province, Xining 810016, China; State Key Laboratory of Severe Weather Meteorological Science and Technology & Institute of Artificial Intelligence for Meteorology, Chinese Academy of Meteorological Sciences, Beijing 100081, China; Hebei Key Laboratory of Meteorological Artificial Intelligence, Meteorological Science Modeling Research and Development Center, Xiong’an Institute of Meteorological Artificial Intelligence, Xiong’an New Area 070001, China; School of Computer Science and Technology, Tongji University, Shanghai 201804, China; Department of Mathematical Sciences, Seoul National University, Seoul 08826, Republic of Korea; Artificial Intelligence Innovation and Incubation Institute, Fudan University, Shanghai 200433, China; Artificial Intelligence Innovation and Incubation Institute, Fudan University, Shanghai 200433, China; National Meteorological Center of CMA, Beijing 100081, China; Shanghai Artificial Intelligence Laboratory, Shanghai 200030, China; Shanghai Artificial Intelligence Laboratory, Shanghai 200030, China; Earth System Modelling, School of Engineering and Design, Technical University of Munich, Munich 85521, Germany; Potsdam Institute for Climate Impact Research, Potsdam 14412, Germany; Allen Institute for Artificial Intelligence (Ai2), Seattle WA 98105, USA; Key Laboratory for Semi-Arid Climate Change of the Ministry of Education, College of Atmospheric Sciences, Lanzhou University, Lanzhou 730000, China; Institute of Meteorological Artificial Intelligence Research, Lanzhou University, Lanzhou 730000, China; Environmental Planning Institute, Seoul National University, Seoul 08826, Republic of Korea; Learning the Earth with AI and Physics (LEAP) NSF Science and Technology Center, Columbia University, New York NY 10027, USA; Shanghai Artificial Intelligence Laboratory, Shanghai 200030, China; Department of Earth System Science, Tsinghua University, Beijing 100084, China; Center for Climate and Carbon Cycle Research, Korea Institute of Science and Technology, Seoul 02792, Republic of Korea; School of Computing, Korea Advanced Institute of Science and Technology, Daejeon 34141, Republic of Korea; Center for Climate and Carbon Cycle Research, Korea Institute of Science and Technology, Seoul 02792, Republic of Korea; School of Atmospheric Sciences, Nanjing University, Nanjing 210063, China; State Key Laboratory of Climate System Prediction and Risk Management (CPRM)/Jiangsu Key Laboratory of Intelligent Weather Forecasting and Applications Based on Big Data/ICAR/CIC-FEMD/KLME/ILCEC, School of Artificial Intelligence, Nanjing University of Information Science and Technology, Nanjing 210044, China; Information & Electronics Research Institute, Korea Advanced Institute of Science and Technology, Daejeon 34141, Republic of Korea; School of Computer Science and Technology, Tongji University, Shanghai 201804, China; State Key Laboratory of Climate System Prediction and Risk Management (CPRM)/Jiangsu Key Laboratory of Intelligent Weather Forecasting and Applications Based on Big Data/ICAR/CIC-FEMD/KLME/ILCEC, School of Artificial Intelligence, Nanjing University of Information Science and Technology, Nanjing 210044, China; State Key Laboratory of Climate System Prediction and Risk Management (CPRM)/Jiangsu Key Laboratory of Intelligent Weather Forecasting and Applications Based on Big Data/ICAR/CIC-FEMD/KLME/ILCEC, School of Artificial Intelligence, Nanjing University of Information Science and Technology, Nanjing 210044, China; State Key Laboratory of Climate System Prediction and Risk Management (CPRM)/Jiangsu Key Laboratory of Intelligent Weather Forecasting and Applications Based on Big Data/ICAR/CIC-FEMD/KLME/ILCEC, School of Artificial Intelligence, Nanjing University of Information Science and Technology, Nanjing 210044, China; State Key Laboratory of Climate System Prediction and Risk Management (CPRM)/Jiangsu Key Laboratory of Intelligent Weather Forecasting and Applications Based on Big Data/ICAR/CIC-FEMD/KLME/ILCEC, School of Artificial Intelligence, Nanjing University of Information Science and Technology, Nanjing 210044, China; State Key Laboratory of Climate System Prediction and Risk Management (CPRM)/Jiangsu Key Laboratory of Intelligent Weather Forecasting and Applications Based on Big Data/ICAR/CIC-FEMD/KLME/ILCEC, School of Artificial Intelligence, Nanjing University of Information Science and Technology, Nanjing 210044, China; State Key Laboratory of Climate System Prediction and Risk Management (CPRM)/Jiangsu Key Laboratory of Intelligent Weather Forecasting and Applications Based on Big Data/ICAR/CIC-FEMD/KLME/ILCEC, School of Artificial Intelligence, Nanjing University of Information Science and Technology, Nanjing 210044, China; State Key Laboratory of Numerical Modeling for Atmospheric Sciences and Geophysical Fluid Dynamics, Institute of Atmospheric Physics, Chinese Academy of Sciences, Beijing 100029, China; Department of Computer Science and Technology, Tsinghua University, Beijing 100084, China; State Key Laboratory of Climate System Prediction and Risk Management (CPRM)/Jiangsu Key Laboratory of Intelligent Weather Forecasting and Applications Based on Big Data/ICAR/CIC-FEMD/KLME/ILCEC, School of Artificial Intelligence, Nanjing University of Information Science and Technology, Nanjing 210044, China; Department of Computer Science and Technology, Tsinghua University, Beijing 100084, China; The Commonwealth Scientific and Industrial Research Organisation, Sydney 2113, Australia; University of New South Wales, Sydney 2052, Australia; Jiangxi Climate Center, Nanchang 330096, China; State Key Laboratory of Climate System Prediction and Risk Management (CPRM)/Jiangsu Key Laboratory of Intelligent Weather Forecasting and Applications Based on Big Data/ICAR/CIC-FEMD/KLME/ILCEC, School of Artificial Intelligence, Nanjing University of Information Science and Technology, Nanjing 210044, China; School of Computer Science and Technology, Tongji University, Shanghai 201804, China; Hebei Key Laboratory of Meteorological Artificial Intelligence, Meteorological Science Modeling Research and Development Center, Xiong’an Institute of Meteorological Artificial Intelligence, Xiong’an New Area 070001, China; Public Meteorological Service Centre, China Meteorological Administration, Beijing 100081, China; State Key Laboratory of Climate System Prediction and Risk Management (CPRM)/Jiangsu Key Laboratory of Intelligent Weather Forecasting and Applications Based on Big Data/ICAR/CIC-FEMD/KLME/ILCEC, School of Artificial Intelligence, Nanjing University of Information Science and Technology, Nanjing 210044, China

**Keywords:** AI application and challenge, atmosphere–ocean sciences, explainable AI, AI-MIP, AI agent

## Abstract

Artificial intelligence (AI) is rapidly transforming Earth science, offering unprecedented capabilities to tackle the most pressing challenges in the field. This work explores significant advances and emerging challenges across the AI for atmosphere–ocean sciences, while outlining critical ways forward. We review deep-learning methods and their application in weather and climate forecasting, which outperforms dynamical models in accuracy and computational efficiency. The role of AI in detecting complex phenomena, enhancing data assimilation and reconstruction, bias correction and downscaling coarse model outputs is also examined. However, the ‘black-box’ nature of complex AI models necessitates a focus on explainable AI to build trust and extract mechanistic insight. The most promising path forward is identified as the development of hybrid physics–AI modeling, which integrates the data-driven power of AI with the foundational constraints of physical laws to ensure generalizability and causal consistency. A new framework for AI-based model intercomparison is essential for rigorous benchmark performance. Finally, we contextualize these technical developments by discussing the usefulness and applicability of AI to society, including the improvement of multi-hazard early-warning systems and green energy production. We conclude by envisioning the future of AI agents for Earth science—autonomous, goal-oriented systems capable of designing and running experiments, generating and testing hypotheses, and learning dynamics from multisource data. This synthesis underscores that AI is not merely a tool, but a paradigm shift, which will significantly improve how we understand and adapt to a changing climate.

## Teaser

This review explores how AI is transforming atmosphere-ocean sciences, from improved forecasts to trustworthy hybrid systems. It outlines a paradigm shift toward autonomous AI agents that could one day drive scientific discovery.

## INTRODUCTION

Atmospheric and oceanic sciences have progressed through the combined supports of monitoring, theoretical analysis and numerical modeling over the past century. Intensified observations reveal major atmosphere and ocean phenomena—frontal systems, eddies, waves and large‑scale circulations—and make it possible to trace their complete life cycles. Meanwhile, advances in fluid dynamics and thermodynamics have paved the way to a solid physical framework for understanding atmospheric and oceanic patterns [1–4].

To forecast them, numerical weather- and ocean-prediction systems have assembled all available data from *in situ* and remote observations into gridded initial conditions. High‑performance computers then solve the governing equations to simulate the evolution of atmospheric and oceanic processes on timescales spanning from hours to decades [5,6]. While this classic paradigm has provided a reliable approach for understanding and predicting atmosphere and ocean phenomena, three long-standing challenges still remain. First, data-assimilation (DA) methods continue to struggle with the sheer volume and heterogeneity of expanded observations. Second, uncertainties in subgrid process parameterizations give rise to systematic biases that worsen in extreme-event simulations. Third, the exponentially increased computational cost for high‑resolution ensemble forecasting has prevented further model improvements.

Artificial intelligence (AI)—more precisely, deep learning (DL)—now offers a new promising and complementary approach to mitigate these challenges. By directly mining nonlinear relationships from large datasets, AI models reconstruct missing or finer-resolution observations in seconds [7], detect and track critical phenomena [8] and deliver global forecasts on hourly, days, seasonal and even decadal timescales [9–11], with their performance often exceeding those of current dynamical models.

However, several key issues should be resolved before AI can be fully integrated into atmospheric and oceanic sciences. For instance, existing techniques for incorporating ever larger and more diverse observation streams into AI models are still primitive, limiting their performance in real-time forecasts. Most of the purely data-driven AI architectures lack fundamental conservation laws and dynamical balances, which leads to their low explainability and undermines their reliability in dealing with unprecedented or extreme conditions [12,13]. In addition, global AI forecast models also suffer systematic biases in their temporal and spatial structures [12] and tools to trace forecast errors back to model components or initial inputs have not yet matured. It is also unclear whether a single AI framework can cover the entire spectrum of forecast horizons from immediate nowcasting to seasonal–decadal predictions as well as climate projections [13], which dynamical models can fully provide.

This review article summarizes recent advances and challenges in broad AI applications in atmosphere–ocean sciences along with potential ways forward. After giving a succinct introduction of DL and its key difference from traditional methods in the second section, the third and fourth sections focus on the AI applications for the detection of atmospheric, land and oceanic phenomena, data reconstruction and assimilation. The fifth section reviews AI performance across different timescales on weather–subseasonal to interannual–decadal forecasts, along with the evolution of large AI models, while the sixth section focuses on the bias correction and downscaling of dynamical model products. Concerning the intrinsic defects of DL, the seventh to ninth sections review possible approaches to improving their transparency, reliability and performance evaluation, focusing on eXplainable AI (XAI), hybrid physics–AI modeling and AI-based model intercomparison. The tenth section briefly discusses how AI-based models can better serve society. The final section presents the latest progress in AI agents for climate science, which provides a profound step toward a humans–AI collaborative paradigm for better confronting our challenges.

## AI DL VERSUS PHYSICS-BASED AND CLASSIC STATISTICAL METHODS

Scientists have long relied on two main categories of approaches to understand and predict weather and climate processes as well as their variabilities, i.e. physics-based and statistical models [14]. These approaches have collectively formed a robust analytical framework that has advanced our understanding of weather and climate systems. Given the established efficacy of these classical methods, it has become essential to examine the unique capabilities that DL brings to atmosphere–ocean sciences and how these methods complement or enhance existing tools.

### Physics-based models

Physics-based models form the cornerstone of Earth science, grounded in fundamental principles such as conservation laws and thermodynamics. However, they exhibit systematic biases stemming from the closure problem, in which unresolved small-scale processes must be approximated through parameterizations [15]. Current schemes often rely on empirical relationships derived from limited observations or high-resolution simulations, introducing substantial uncertainties and context-dependent biases that vary across weather–climate regimes and scales. These biases propagate through the system, impacting both short-term weather forecasts and long-term climate prediction/projection.

While physics-based models excel at the step-by-step integration of governing equations, deficiencies in representing small-scale and slowly evolving physical processes often limit their ability to capture emergent patterns across multiple spatiotemporal scales. The reducible structures evident in the phase space of these dynamical systems suggest that pure physical integration—despite its mechanistic rigor—may not be optimal for all applications. Data-driven methods can complement traditional modeling by directly identifying and exploiting these patterns, offering enhanced insights into complex phenomena [16].

### Statistical models

While the weather and climate are governed by a chaotic, high-dimensional, multiscale dynamical system, the atmosphere–ocean sciences have traditionally relied on several key statistical approaches, focusing on extracting dominant patterns from observed data. Using techniques such as linear regression, compositional analysis and time-series analysis, these models reduce dimensionality and analyse spectra to navigate the complexity of the system. Core to this approach is the incorporation of randomness to represent chaotic natural features, resulting in a framework that is probabilistic and largely agnostic toward causality. However, these statistical approaches typically rely on prior knowledge via a parametric prior (e.g. Gaussian distributions), while the high-dimensional, multiscale and chaotic nature of weather and climate systems often violates these assumptions.

### DL

DL represents a particularly powerful branch of machine learning (ML) that employs hierarchical layered transformations and abstractions [17]. When data and computation are abundant, DL demonstrates exceptional advantages through several key innovations in the learning process as follows:


(1) \begin{eqnarray*}
\mathit{Learning} &=& \mathit{Representation} + \mathit{Evaluation}\nonumber\\
&&+\, \mathit{Optimization},
\end{eqnarray*}

where ‘Representation’ means to choose how to transform raw data into useful features and select a model architecture that defines the hypothesis space that the learner can explore [18,19]. ‘Evaluation’ means to specify the function that defines ‘good’ performance and steer the behavior of the model during training. ‘Optimization’ means the process that uses data-driven algorithms to adjust parameters to minimize loss and achieve the defined objective. Note that representation (including both feature representation and function representation), evaluation and optimization are not treated separately in DL. Below we highlight how modern DL combines these aspects synergistically for superior performance.

DL revolutionizes function representation by automating feature discovery, in which models adaptively extract task-relevant patterns that optimize specified loss functions, often uncovering insights beyond human-engineered features [20]. This benefit is particularly evident given abundant, high-quality training data. Hierarchical architectures enable the progressive composition of low-level features into abstract, invariant representations, facilitating the nonlinear modeling of complex phenomena and offering superior predictive power over shallow methods. Depth plays a pivotal role in enhancing inductive biases that leverage inherent data symmetries and structures in deep architectures. For instance, convolutional neural networks (CNNs) embed translational invariance for processing gridded data [21]; recurrent neural networks (RNNs) such as long short-term memory (LSTM) capture temporal dependencies in time-series forecasting [22–24]; graph neural networks (GNNs) are utilized to model the relational topologies inherent in weather and climate networks [16,25]; neural operators incorporate spectral mappings for partial differential equation (PDE)-based modeling [26,27]; transformers facilitate global interactions for teleconnections [28]; and hybrid architectures such as convolutional-long short-term memory (Conv-LSTM) integrate multiple biases for spatiotemporal tasks [29]. Beyond discriminative architectures, generative DL approaches—including variational autoencoders (VAEs) [30], generative adversarial networks (GANs) [31] and diffusion models [32,33]—learn to approximate underlying data distributions from samples. In particular, multistep generative models such as diffusion processes, formulated through stochastic differential equations or flow matching [34], progressively capture multiscale spectral structures inherent in data. This hierarchical generation process offers considerable potential for developing foundation models capable of addressing key challenges in DA and seamless prediction across timescales [35].

Evaluation fundamentally revolves around differentiable objectives that enable gradient-based optimization. These include cross-entropy loss for classification tasks; probabilistic losses such as those parameterizing output distributions for regression with uncertainty quantification; generative paradigms such as VAEs [30], GANs [31], diffusion models [32,33] and flow matching [36] for learning high-dimensional distributions; and reward maximization for reinforcement learning [37].

## AI-BASED PHENOMENA MONITORING, DETECTION, CLASSIFICATION AND SEGMENTATION

The identification and tracking of phenomena have traditionally relied on physical models or empirically defined threshold-based methods. However, these conventional approaches face significant limitations in multimodal data coupling, fine-scale feature extraction and heterogeneous data fusion. AI-based methods overcome these constraints by data mining representations, eliminating the dependence on manually crafted rules and thresholds. This advancement enables automated, multiscale information extraction—from object-level detection to pixel-level segmentation—and marks a paradigm shift in the dynamic monitoring and mechanistic understanding of multiscale phenomena [38,39].

### Atmosphere-phenomena detection

**Figure 1.**
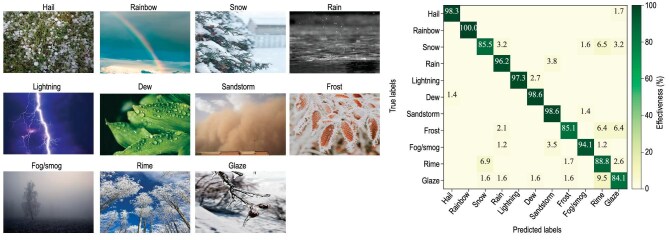
Representative examples of 11 weather-phenomena images (left panel) and the effectiveness of a CNN model evaluated by using the confusion matrix (right panel) [39].

AI-based methods have achieved significant progress in monitoring and analysing various atmosphere phenomena (Fig. 1), including hail, frost, rainbow and smog. In addition, based on remote sensing data, DL models were built to detect and classify tropical cyclones (TCs) and estimate their intensities [40], providing pixel-level identification of TCs and atmospheric rivers [41] and accurate precipitation-type classification [42], achieving superior accuracy to those of traditional methods.

### Land-phenomena detection

**Figure 2.**
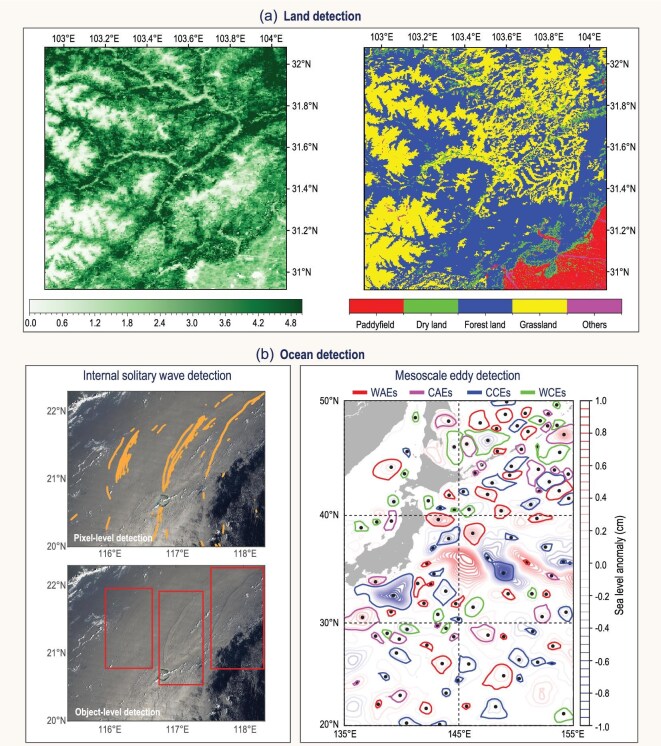
(a) AI-based land detection. Leaf-area index (left) derived by using an artificial neural network from MODIS and LULC classification (right) by using CNNs from Landsat, shown over a region in Sichuan Province, China (30.92°–32.08°N, 102.92°–104.08°E) on 1 June 2020. (b) AI-based ocean detection. Internal solitary waves (ISWs, left) detected at multiple levels: pixel-level and object-level detections. AI-based eddy-detection map (right) categorizes mesoscale eddies into four classes: warm anticyclonic eddies (WAEs, red contours), cold anticyclonic eddies (CAEs, magenta contours), cold cyclonic eddies (CCEs, blue contours) and warm cyclonic eddies (WCEs, green contours).

Key applications include the monitoring of land-use and land-cover (LULC) changes, surface albedo and temperature, soil moisture, evapotranspiration, water reservoirs, cryosphere dynamics (glaciers and ice sheets), snow, vegetation and crop growth, as well as related natural hazards such as bushfires, landslides, droughts and river flooding [43]. Among these, LULC is particularly critical as a primary input for the land-surface component of Earth-system models (ESMs), supplying essential information on vegetation types, urban extents and crop land [44]. Despite the abundance of satellite-derived LULC products, inconsistencies in classifications hinder their direct application in ESMs. Addressing these discrepancies through data harmonization remains a key challenge for improving the accuracy and utility of LULC datasets. Another key challenge involves harnessing DL to reconstruct historical trends and project the future dynamics of land-surface variables—such as Land Use/Land Cover Change and the leaf-area index (Fig. 2a)—beyond the temporal coverage of available satellite observations, especially at high resolution.

### Ocean-phenomena detection

With AI and multisource remote sensing data—such as infrared, microwave and synthetic aperture radar, complex oceanic processes are investigated with unprecedented precision and efficiency [45]. For instance, a number of DL architectures were constructed to successfully detect internal solitary waves at both the object level (bounding boxes) and the pixel level (wave-crest masks) (Fig. 2b, left panel) [46], achieving robust cross-sensor performance and providing a valuable data foundation for forecasting and dynamic analysis. A residual U-Net architecture was also employed to identify four classes of mesoscale eddies—warm-core anticyclones, cold-core anticyclones, cold-core cyclones and warm-core cyclones [47]—uncovering thermodynamically ‘abnormal’ eddies often missed when using traditional techniques (Fig. 2b, right panel).

## DATA RECONSTRUCTION AND ASSIMILATION

**Figure 3.**
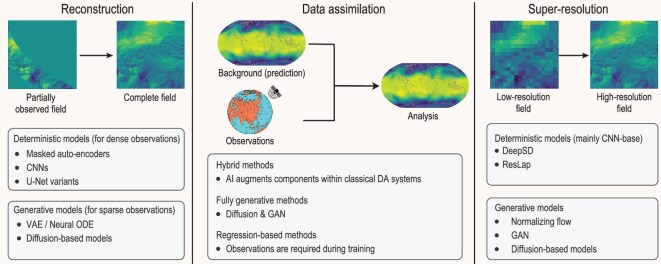
The three core scenarios of AI approaches for atmospheric and oceanic data processing.

Obtaining high-resolution and physically consistent gridded fields is fundamental for atmosphere–ocean sciences. Applications such as regional weather and climate analyses, Earth-system and extreme-event prediction require accurate spatiotemporal representations of key state variables. Three core scenarios arise in practice: reconstruction from partially observed fields, super-resolution from coarse-grid simulations and DA integrated from model forecasts and observations (Fig. 3). Each presents distinct challenges and has spurred the development of increasingly sophisticated AI approaches.

### Data reconstruction

Traditional methods, such as optimal interpolation or empirical orthogonal functions, rely on strong assumptions (e.g. linearity, stationarity, Gaussianity) and therefore often fail to recover spatiotemporally consistent structures. DL offers a more flexible alternative, especially in cases with relatively dense or structured observations. Deterministic supervised models learn direct mappings from masked fields to complete fields by using architectures such as masked autoencoders [48], partial convolutional networks [49] and U-Net variants with uncertainty estimation [50]. GANs have also been applied for inpainting and radar recovery [51,52]. Recent improvements include the use of auxiliary physical variables (e.g. wind, chlorophyll) [50] and transfer learning from pretrained climate models [7,53]. The LaMa DL model has recently been shown to outperform existing statistical and DL approaches for reconstructing global historical temperature fields [54].

More ill-posed settings arise when observations are sparse, irregular or multimodal (e.g. paleoclimate proxies, isolated sensors). They require generative models that approximate the distribution of plausible states. Most admit a two-stage paradigm: learning a prior via VAEs or neural ordinary differential equations, followed by posterior sampling conditioned on sparse inputs [55]. Diffusion-based models extend this framework by incorporating observations during sampling via score-based guidance, as in S3GM [56] and related approaches [57].

### DA

DA aims to generate dynamically consistent analyses by combining background forecasts with observations. Traditional techniques, such as 3DVar, 4DVar and ensemble Kalman filters, are grounded in physical principles, but depend heavily on sample-estimated background-error covariances, limiting the flexibility in non-Gaussian or nonlinear settings. Due to the similarity between optimizing DL networks and solving the analysis equation in variational DA methods—both relying on the gradient calculation of the loss function—it has been widely discussed that DL networks could mimic the DA process [58–60].

AI-based DA approaches fall into two paradigms: hybrid and fully generative. In hybrid methods, AI augments specific components within classical DA systems. A first class of hybrid approaches focuses on shared DA components that are common to both the 3DVar and the 4DVar frameworks. For instance, neural networks have been used to model background-error covariances [61] and satellite radiance observation operators [62], enabling more flexible and nonlinear representations while remaining compatible with variational DA formulations. A second class of hybrid approaches specifically targets challenges that are more prominent in 4DVar, such as flow-dependent background errors, adjoint models and the high computational cost of optimization over assimilation windows. AI-based methods have been proposed to improve flow-dependency modeling [63,64], accelerate optimization schemes [63] and approximate adjoint models [65], thereby alleviating the computational and optimization difficulties inherent in 4DVar. In ensemble-based settings, AI has also been applied to improve Kalman gain estimation [66], covariance localization [67] and particle filter efficiency through score-based sampling [68,69]. Latent-space DA, which embeds system states in lower-dimensional manifolds, is also gaining traction [70–72]. However, fully data-driven DA methods are currently more often explored in 3DVar-like settings and their extension to 4DVar, in which flow-dependent dynamics play a central role, remains an open research challenge.

In the fully generative paradigm, AI models learn the posterior distribution of analysis fields directly from the paired training samples of forecasts, observations and analysis states. The U-Net architecture using fusion modules successfully assimilates satellite observations [73–75]. Diffusion models have become the dominant approach due to their ability to capture complex priors and incorporate observations through conditioning or score-based guidance [57,76,77]. GAN-based methods have also been explored [74,78,79], though diffusion models are now preferred due to their greater stability and physical consistency.

By directly using the automatic differentiation algorithm embedded in the AI-based DA, targeted observations that identify the most sensitive regions for improving forecast skill can be detected [80–82], thereby enhancing the overall quality of the AI-based DA.

## AI PREDICTIONS AT DIFFERENT TIMESCALES

DL-based forecast models have begun to outperform current dynamical models, particularly in predicting atmospheric variations along with the occurrence/intensity of extreme events in weather and subseasonal timescales by learning complex, high-dimensional and nonlinear relationships, thereby mitigating forecast errors that often arise from simplifications in traditional physical parameterizations. By modeling land, ocean and sea-ice components and incorporating their relationship with the anthropogenic factors, DL models are anticipated to yield substantial improvements at longer climate timescales. Further advancements in DL methodologies, combined with the integration of physics-informed and meta-learning approaches, can help to address the data-scarcity and ‘black-box’ issues in building data-driven weather and climate models. These innovations enable learning from limited data and contribute to the more effective optimization of DL models.

### Nowcasting

Precipitation nowcasting—high-resolution forecasting of precipitation up to several hours ahead—supports the socioeconomic needs of many sectors. To leverage the advantages of DL models in terms of both predictive accuracy and computational efficiency, DL approaches have been introduced that directly predict future rainfall rates from radar observations with high spatiotemporal frequencies (1-km horizontal resolution with a temporal range from 5 min to 1 h). Although early stages of DL models for precipitation nowcasting accurately predict low-intensity rainfall, their operational utility remains limited due to the blurry nowcasts at longer lead times, resulting in poor performance in predicting heavy-precipitation events [83]. Recent DL models for probabilistic precipitation nowcasting from radar data tend to address these challenges by adopting stochastic generative models [84]. The unnatural motion and intensity in the predicted precipitation are mitigated by introducing an advection-based evolution network that incorporates horizontal-wind information [85]. In MetNet-3, precipitation-related variables from *in situ* observations are further utilized [86]. It achieves a realistic high-resolution structure of precipitation from sparse observations by introducing a process called densification. Namely, certain portions of station data are randomly dropped from the input of the network during training, while keeping these observations as targets, and the model is evaluated both through spatial generalization to unseen stations and through hyperlocal performance at observational locations.

### Weather to subseasonal forecasts

**Figure 4.**
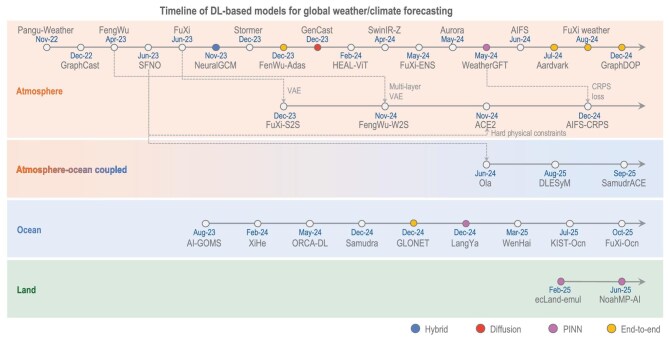
Timeline of DL-based state-of-the-art models for global weather and climate forecasts. Dates indicate the initial release date of each model, and AE, CRPS and PINN are abbreviations for autoencoder, continuous ranked probability score and physics-informed neural network (including physics-guided neural network), respectively.

In addition to regional DL-based models with a few kilometers’ resolution for precipitation nowcasting [87], various DL-based global models have been developed and have achieved superior accuracy in forecasting air temperature, humidity, geopotential heights and horizontal winds, by leveraging historical reanalysis datasets [9,88–98] (Fig. 4). Pangu-Weather based on Swin-Transformer architecture reported 10% lower root mean square errors (RMSEs) in predicting key tropospheric and surface atmospheric variables than the operational integrated forecasting system (IFS) of European Centre for Medium-Range Weather Forecasts (ECMWF), one of the most accurate operational dynamical model-based forecast systems [99]. GraphCast, a GNNs-based model, significantly outperforms the IFS on 90% of the verification targets and supports better prediction of severe events, including tropical cyclone tracking and atmospheric rivers [89]. A spherical multigrid neural operator that integrates spherical harmonic convolution and a spherical Fourier neural operator (SFNO) achieves a significant improvement in predicting lower and middle tropospheric variables with a lead of ≤7 days [100].

The capabilities of DL-based atmospheric models are further extended to cover broader forecasting objectives and longer time horizons. To estimate the uncertainty induced by various atmospheric instabilities, which allows a nuanced interpretation of model outputs and enables users to facilitate robust and risk-aware strategies, DL-based models such as FengWu, FuXi, ArchesWeather and GenCast incorporate diffusion-based generative algorithms into the deterministic DL architectures, harnessing the strengths of generative models [101–104]. Diffusion-based approaches and a noise-injection strategy with model ensembles [105] also effectively mitigate the blurry issue, enhance their ability to predict extreme-weather events (e.g. heatwaves) and improve uncertainty estimation. An integration of diverse data sources enhances the prediction accuracy of the data-driven model by utilizing *in situ* and remote sensing measurements as well as reanalysis [106,107]. Diffusion-based approaches such as generative assimilation and prediction (GAP) have also been shown to be able to unify DA and weather prediction from medium range to seasonal cycle in one framework [35].

To extend forecast durations to subseasonal timescales, a common strategy is to reduce temporal resolution to prevent the accumulation of errors during the iterative forecasts [104,108]. Another way is to construct a loss as a function of forecasts on the subseasonal lead times. There is a multistep loss method in which predictions are performed repeatedly over multiple steps and the loss at all steps is calculated to update the model parameters [89]. For example, the training process of FuXi-S2S follows an autoregressive training regime and a curriculum training schedule, incrementally increasing the number of autoregressive steps from 1 to 17 days’ lead [109]. As a result, FuXi-S2S achieves skillful daily mean predictions for ≤42 days and significantly extends the predictive skill for Madden–Julian oscillation (MJO). In a similar manner, FengWu applied a replay buffer mechanism, which stores the predicted results from previous optimization iterations and uses them as the input for the model for subsequent iterations. As it mimics the intermediate input error during the autoregressive inference stage, it is known to be beneficial for long-lead forecasts with efficient computation and memory [101]. The probability-based loss function in Artificial Intelligence Forecasting System-Continuous Ranked Probability Score contributes to capturing the subseasonal variability in the atmospheric system while preserving the original temporal frequency [96]. The GAP framework also shows remarkable skill at subseasonal scales, including MJO prediction [35].

Enhancing the generalization and physical consistency of DL-based models, NeuralGCM combines a simplified, differentiable atmospheric dynamical core with AI-based physical parameterizations [13]. This hybrid architecture preserves the integrity of large-scale fluid dynamics while allowing the learned components to simulate subgrid processes. If these DL-based parameterizations are modularized and categorized (e.g. NeuralGCM with a precipitation scheme [110]), the entire modeling becomes more transparent and interpretable, thus providing clearer guidance for future model development.

### Seasonal-to-interannual forecasts

**Figure 5.**
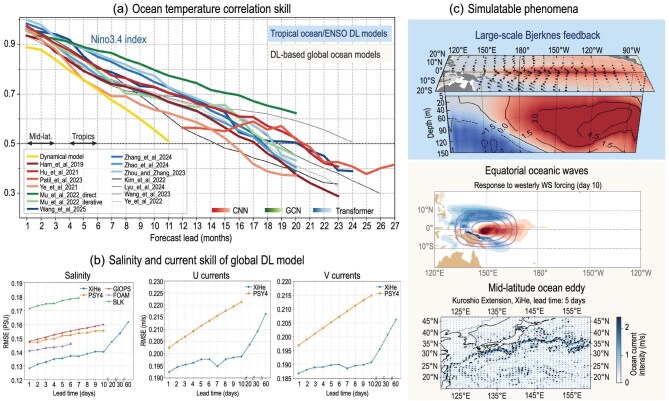
(a) Correlation skill of sea-surface temperature (SST) using various DL-based models. Each model is represented by a line showing the prediction skill of the Niño3.4 SST index, and models are grouped by their base DL architecture (CNN-based, graph convolutional network-based, or transformer-based). Arrows at the correlation threshold of 0.5 indicate the lead-time ranges over which the correlation skill exceeds 0.5 for predictions over tropical and mid-latitude regions using DL-based global ocean models. (b) RMSE of the global salinity and currents of the DL-based XiHe model with four operational numerical models (adopted from [111]). (c) Example of simulatable phenomena of DL-based model targeted to predict tropical ocean variabilities by using monthly fields (upper panel) and DL-based global ocean models (lower panel).

Given that oceanic processes are the primary drivers of climate variability on interannual to decadal timescales, data-driven global ocean models are expected to extend the predictive capabilities of current DL models to longer timescales [11,111]. Various DL models that aimed to predict dominant interannual variability in the tropical Pacific, namely El Niño Southern Oscillation (ENSO), by using architectures such as Vision-Transformer (ViT) [112], convolutional block attention modules [113] and graph convolutions [114] have extended predictive lead times by ≤2 years, thereby surpassing the performance of most advanced dynamical models and a pioneering CNN-based DL model [10] (Fig. 5a). A modified version of the aforementioned DL models has shown promising results in predicting dominant oceanic variabilities in the Indian Ocean [115], the tropical Atlantic Ocean [116,117] and Arctic sea ice [118,119], despite these regions being traditionally considered as less predictable due to shorter-lived and weaker variability [115].

To improve the explainability and applicability of DL models, 3D data-driven ocean models have been developed, enabling more accurate simulation and prediction of dominant interannual and decadal variability with systematically reduced biases compared with those of the state-of-the-art dynamical models (Fig. 5b). DL ocean models, based on Swin-Transformer architectures [28], can resolve oceanic eddies [76] and tropical basin-scale atmosphere–ocean coupled processes [112], capturing both local and global atmospheric [120,121] and oceanic connections [111] (Fig. 5c). It is demonstrated that an SFNO-based [100] coupled atmosphere–ocean model successfully reproduces ENSO-related large-scale Bjerknes feedbacks and associated global atmospheric teleconnection patterns [122]. Through idealized experiments, it is further demonstrated that the DL ocean model is able to simulate realistic 3D thermodynamic responses associated with the equatorial oceanic waves (i.e. Kelvin and Rossby waves) driven by atmospheric wind forcings [11].

By taking the leverage of the DL model to keep any constraints by minimizing the loss function with the differential algorithm, a specified loss can be designed to seek the fast-growing perturbations, which is known to be optimal for ensemble forecasts [123]. As the fast-growing perturbations represent the initial patterns that most sensitively influence the target phenomenon, they can also be interpreted as a powerful tool for identifying optimal precursors and for conducting predictability studies [123–126].

To compensate for limited samples of data, DL models often incorporate synthetic training data from multidecadal simulations of dynamical climate models [10,11,127]. However, similar approaches have proven to be less effective in predicting mid-latitude climate variability [128,129]. This is primarily due to the limited reliability of dynamical model simulations in these regions, stemming from the complexity of their driving mechanisms [130], resulting in a lower signal-to-noise ratio in forecasts [131]. Instead of using simulations of dynamical climate models, meta-learning algorithms facilitate the rapid adaptation of DL models to new tasks by using limited data [132], making it possible to perform skillful mid-latitude climate forecasting based solely on the limited number of observational records [133,134].

### Decadal prediction and climate projection

Climate projection simulates the impacts of varying external forcings, such as greenhouse gases and aerosols, over several decades under various scenarios to inform future policy decisions. AI-based global models typically adopt an autoregressive forecasting approach that performs iterative predictions at intervals ranging from as short as 1 h to as long as 1 month [11,13,100,135]. Consequently, conducting a 100-year simulation requires an autoregressive forecast that repeats ≥10 000 steps. Although data-driven models can qualitatively reproduce realistic dynamical properties across a certain number of time steps [136], errors can accumulate over successive prediction steps if fundamental physical constraints (e.g. conservation of energy) are violated. Moreover, concerns remain about whether AI models can capture climate responses to radiative forcing changes over multidecadal periods that far exceed their prediction timesteps.

To prevent physical violations and enable stable long-term simulations, AI models have been augmented with domain knowledge (i) by embedding physical constraints directly into their architecture and (ii) by creating a hybrid physics–AI model. Physics-informed neural networks apply soft physical constraints through loss-function penalties [137,138], while hard physical constraints enforce conservation laws through architectural design [135,138,139]. Examples include constraints from the momentum equation, continuity equation [140], thermodynamic relations [139], mass-conservation equation [140] and moisture conservation [135]. Sensitivity experiments have identified moisture processes as the primary cause of collapse in long-term simulations, suggesting that constraining moisture conservation is possibly more critical than constraining other physical relations [141–143]. Accordingly, enforcing global moisture conservation and condensation constraints was shown to substantially enhance the stability of long-term climate simulations [135,144]. Notably, it was demonstrated that, even when inference intervals (e.g. 6 h or 1 day) are short relative to the timescales of CO_2_ forcing, AI models can still learn and reflect CO_2_‑induced climate responses [135].

Hybrid physics–AI models further address physical inconsistencies and support stable long-term integrations by deploying AI primarily in a supportive role. Key implementations include: integrating a dynamical core or process-based model with a neural network-based learnable physics module [13,141,144–146], adapting differentiable modeling frameworks to jointly optimize physical and neural network components [43] and employing AI for systematic bias correction in dynamical model forecasts [147].

Despite these advances, NeuralGCM still yields unrealistic outputs under extreme-warming scenarios (+4 K) [13], indicating limits to the extrapolation beyond training regimes. Climate‑invariant transformations (e.g. from specific humidity to relative humidity) to stabilize probability density functions across regimes [138] and deep stable learning techniques that remove spurious correlations to focus on intrinsic features under distribution shifts [148] can help mitigate the climate extrapolation challenge under unseen extreme conditions.

Decadal climate prediction bridges seasonal forecasting and long‑term projections by targeting 1–10 years of variability and hence requires the representation of internal modes such as the ENSO, Pacific Decadal Oscillation (PDO) and Atlantic Multi-decadal Oscillation (AMO) [149–151]. Map-to-index AI models targeting specific decadal variability, such as PDO and AMO, have achieved skillful multiyear to multidecadal predictions [152,153]. Beyond this, recent map-to-map AI ocean models have demonstrated robust predictive skills for decadal variabilities for up to multiple years [11].

## POST-PROCESSING OF DYNAMICAL MODEL PRODUCTS

To mitigate the limitations of numerical weather predictions (NWPs) and climate forecasts of dynamical models induced by coarse resolutions, non-perfect parameterization schemes and initializations [154], post-processing approaches have been widely applied, such as correcting bias and the downscaling of temperature, precipitation and wind forecasts [155]. Recently, AI has played a key role in post-processing due to its ability to depict nonlinear relationships and it efficiently improves the prediction skills of dynamical models at different scales (Table 1).

**Table 1.** Bias correction for dynamical model prediction at different timescales.

Timescale	Variables	Methods	Advantage	References
Weather forecast	Wind	ML and stacking methods	Effectively combine different foundational models for bias correction	[157]
	Precipitation	DL	Correct the bias in the precipitation prediction through considering different variables	[158]
	Temperature	DL	The performance of bias correction through the DL is better than that of the traditional method	[159]
Climate prediction	Temperature	Adaptive bias correction	Combines state-of-the-art dynamical forecasts with observations using ML	[163]
	Precipitation	DL	Reduced bias in Western Pacific Subtropical High (WPSH) and SST is critical to enhancing forecast accuracy	[160]
	MJO	DL	Blend the dynamical forecasts and observations to improve the accuracy of MJO prediction	[164]
Climate projection	Precipitation	GNNs	Distinguish historical climate simulation samples and observation samples	[167]

### AI bias correction for dynamical weather forecasts

By distilling patterns from vast archives of observations and model outputs, machine-learning techniques have been utilized to compensate for dynamical model biases and generalize across diverse weather and climate regimes, delivering noticeable gains in both accuracy and reliability. Early studies demonstrated that random forests and support-vector regressors, when trained by using a direct-mapping strategy, outperform classical statistical post-processing schemes for short-term wind-speed forecasts [156]. Furthermore, ensemble strategies such as stacking generalization have attracted growing interest for their ability to blend complementary strengths and suppress residual errors [157]. Similar AI-driven advances are now being extended to precipitation and temperature prediction, signaling a broader paradigm shift in operational forecasts [158,159] (Table 1).

### AI bias correction for dynamical climate simulation and prediction

AI approaches have also been applied to correct biases in dynamical climate prediction. Compared with weather forecasts, bias correction with AI in climate prediction is more difficult due to the lack of sufficient samples [155]. Nevertheless, ML and DL have helped to improve the seasonal prediction skills for precipitation over China [160], displaying obvious advantages over the conventional quantile mapping method [161]. The results suggest that reduced biases in predicting the Western Pacific Subtropical High and sea-surface temperatures is critical to enhancing the precipitation forecast accuracy [160], consistently with the well-recognized mechanism. Data-driven methods also help to optimize the weights of ensemble prediction [162]. Regarding the subseasonal to seasonal (S2S) prediction, in which MJO provides a primary predictability source but has not yet been well predicted in dynamical forecasting systems, the ML and DL bias-correction methods have been applied to blend the dynamical forecasts and observations to improve the accuracy of temperature, precipitation and MJO prediction [163,164] (Table 1).

The ML and DL methods have also been utilized to improve long-term climate simulations made by dynamical models. A spatial-based 2D Conv-LSTM network was proposed to correct the bias in spatial dynamics and precipitation products from regional climate models (RCMs) [165]. Furthermore, online corrections can be integrated into a model running cycle as well, allowing subsequent cycles to benefit from previous improvements. For instance, CNN online bias-reduction models were developed to create a corrective model parameterization, improving the accuracy of precipitation and MJO simulations [166]. To mitigate the weakness that existing approaches usually focus on limited low-order statistics or break the spatiotemporal consistency of the target variable, a regularized adversarial domain adaptation methodology was developed to overcome these deficiencies, enhancing the efficient identification and correction of climate model biases [167] (Table 1).

### AI downscaling for dynamical model outputs

Traditional approaches include interpolation (e.g. bilinear, spline), statistical disaggregation and downscaling based on climatological regression and analog matching. However, these methods either assume fixed spatial relationships and thus exhibit limited generalizability or suffer from high computational costs. AI-based downscaling aims to enhance the spatial resolution of geophysical fields by learning mappings from coarse-resolution simulations to high-resolution representations.

Recent advances in AI have enabled more flexible and data-driven resolution enhancement by learning nonlinear mappings from coarse to fine scales (Fig. 3). Deterministic supervised models, such as DeepSD [168] and ResLap [169], employ CNNs for efficient upscaling, but often underestimate fine-scale variability and associated uncertainties. To address these limitations, generative models have been increasingly adopted to sample from the conditional distribution of high-resolution fields. Representative approaches include normalizing flows [170], GAN-based architectures [171,172], progressive and multiscale GAN variants [173,174], as well as more recent diffusion and consistency models [175–178]. Additional architectural components, such as attention mechanisms, have further improved performance, particularly in capturing spatial heterogeneity and extreme events [179]. These AI-based super-resolution and downscaling methods have been successfully applied across a wide range of weather and climate applications, including precipitation and temperature downscaling, climate field reconstruction and the refinement of dynamical model-generated products [180–182].

## EXPLAINABLE AI

**Figure 6.**
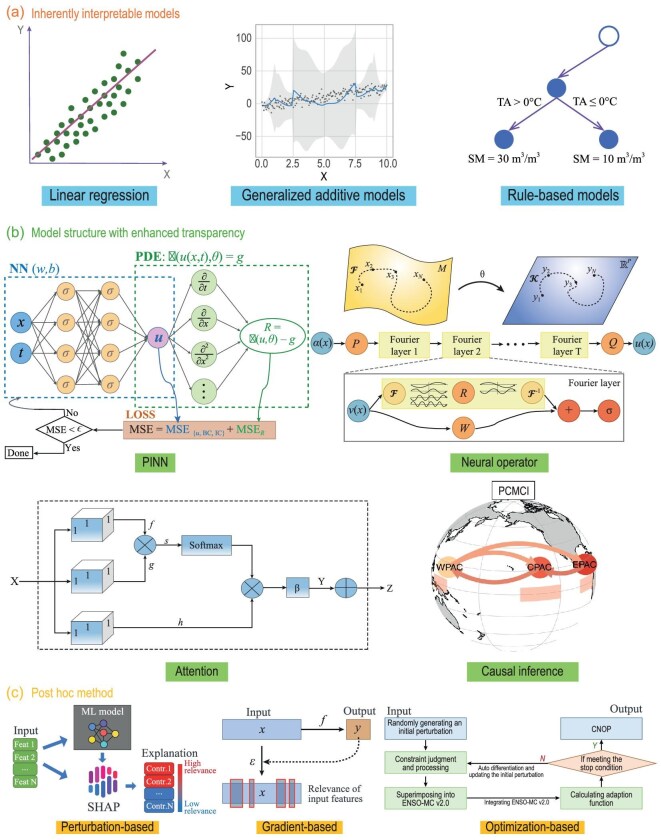
Three types of XAI technologies, including (a) inherently interpretable models, (b) model structures with enhanced transparency and (c) post-hoc method.

Though AI has been widely used in atmosphere–ocean sciences, the intrinsic ‘black-box’ nature of AI raises an indispensable double crisis [183], i.e. it completely shields the decision-making process from inferences and shakes the foundation of the scientific community’s trust in AI to a large extent. To address this challenge, XAI technology [184] attempts to answer fundamental questions, such as ‘How does AI effectively extract physical laws from data?’, by systematically dismantling the key components of AI models. In principle, applications of XAI in atmosphere–ocean modeling can be classified into three types (Fig. 6) [185,186].

First, inherently interpretable AI models such as linear regression, generalized additive models, decision trees and rule-based systems are commonly used due to their ability to display the way in which decisions are made (Fig. 6a). However, they often sacrifice predictive performance compared with more complex models such as deep neural networks [187]. Note that these types of methods are sometimes considered not as XAI, but as so-called interpretable AI.

Second, well-developed geoscience theories and physical mechanisms can guide AI modeling for better feature extraction from large datasets, which makes the model more explainable. Based on many successes, this has gradually become a modeling statute (Fig. 6b). Physics-informed neural networks are recognized as one of the most explainable architectures and they directly incorporate PDEs into neural layers and loss functions [188]. Attention layers are beneficial in revealing element-level characteristics, such as spatiotemporal and variable domains [189–191], and neural operators [192,193] facilitate the capture of variability at different temporal frequencies. It is also noteworthy that causal neural networks, which combine the rigorous causal inference theory with AI modeling [194,195], are effective tools for mining knowledge from data. Time-lag or teleconnection information between multiple physical variables and processes can be efficiently expressed in the form of causal diagrams.

Third, utilizing mature post-hoc explainable methods in a well-trained AI model can derive significant scientific findings, which helps to make black-box models transparent (Fig. 6c). These methods can be generally grouped into four categories. The first two types of methods are called model-agnostic, while the other two are model-specific. The first type is a surrogate approach. Global surrogates approximate the entire behavior of a black-box model by using inherently interpretable AI models such as decision trees, while local surrogates explain the predictions for individual instances (e.g. local interpretable model-agnostic explanations [196]). The second type is a perturbation-based attribution approach. Shapley additive explanation [197] is the most representative, which effectively detects the contributions of individual features or well-designed feature groups by AI model input modifications [198]. The third type is a gradient-based attribution approach [199] and is the most widely used method for DL models. With the help of the automatic derivation of AI models, one can easily explore the gradient of various objective functions with respect to the input to generalize physical meanings [115,200]. The fourth type is the optimization-based attribution approach, which overcomes the strong assumption of linearity and small perturbation magnitude in the first two types of approaches [201]. Conditional nonlinear optimal perturbation [202] is the most effective method, which takes advantage of the strong nonlinear characteristics of AI models and uses optimization to reveal the initial perturbations that most affect the model skill [125].

It should be emphasized that the effectiveness of the XAI technology is strictly dependent on the high performance of AI models [201]. Physical inconsistency may arise if there are fatal systematic biases in AI models. For example, AI weather-forecasting models trained on present-day climate data exhibit systematic biases, such as a global-mean cold drift, when applied to future warmer climate scenarios, causing physically inconsistent interpretations of climate dynamics [203]. In addition to the structure of AI models, the construction and selection of high-quality data used in AI modeling—such as ensuring relevance, representativeness, physic consistency and fairness—are critical for enhancing the robustness and reliability of explanations derived from XAI. It should be noted that different AI models and different XAI methods may offer inconsistent explanations. As a result, the derived explanations should be carefully examined among different approaches. The application of XAI in Earth-system science is still in its infancy and tremendous efforts are needed to develop co-designed frameworks that integrate domain knowledge, causal inference and human-centered interfaces [186].

## HYBRID PHYSICS–AI MODELING

Traditional NWP systems rely on PDEs that describe atmospheric dynamics, thermodynamics and various physical processes. However, these models are often computationally expensive and sensitive to both initial conditions and model resolution. Meanwhile, purely data-driven models, although fast and flexible, often lack fundamental physical constraints, making their extrapolation to out-of-sample conditions questionable. To address these limitations, hybrid physics–AI methods integrate the best of both worlds: the interpretability and rigor of physical models with the adaptability and predictive power of modern ML.

**Figure 7.**
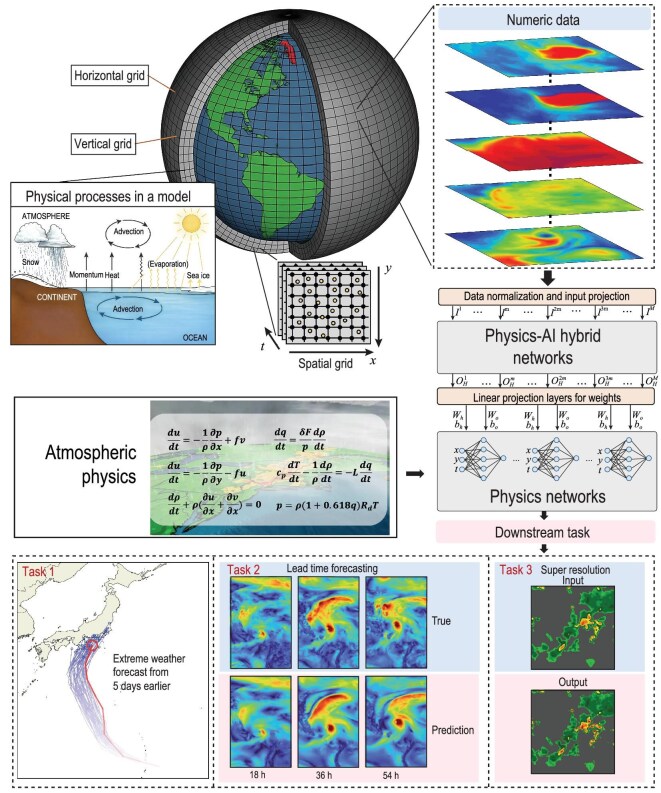
Workflow of the hybrid physics–AI system and results. This figure presents a composite overview of recent state-of-the-art hybrid physics–AI weather-forecasting models, including GenCast, WeatherGFM, DeepPhysiNet and FourCastNet. It integrates representative workflows and selected results from these models to effectively illustrate the architectural structure and core operators of the hybrid modeling approaches. Subfigures are adapted from GenCast [105], DeepPhysiNet [205], and FourCastNet [192] with modifications.

Physics-informed machine learning (PIML) exemplifies this synergy by directly incorporating physical equations into the neural network training process [204,205]. In weather and climate contexts, PIML can embed dynamical and thermodynamical equations into the loss function so that the model learns to satisfy physical constraints while fitting the observed or simulated data [206]. Alternatively, methods to directly modify learned dynamics use stabilization techniques to ensure that conservation laws have been introduced for chaotic dynamics [207]. This explicit blending of data and physics aims to reduce the amount of data required and improve generalization, as the network is guided not just by statistical correlations, but also by known laws of fluid dynamics and thermodynamics (Fig. 7).

AI is now transforming our capability to rapidly and accurately simulate complex multiscale processes, particularly in weather and climate prediction. Previous AI methodologies, such as sparse representation, RNNs, reservoir computing and related approaches, have demonstrated significant potential for modeling dynamical systems [208,209]. Recent advancements, particularly neural operators, further enhance this potential by providing a generalized framework for interpreting and modeling these complex phenomena at finer scales, resulting in high-fidelity predictions. Neural operators generalize traditional neural networks by learning operators—mappings between input and output functions defined over continuous domains [27,152]. Unlike traditional numerical approaches, neural operators are not limited to explicitly solving PDEs; instead, they learn representations directly from data, capturing underlying physics implicitly through observations or simulations. That is, neural operators encompass many data-driven weather and climate models, inherently learning mappings between physical states across various temporal and spatial scales, and therefore can avoid inaccuracies resulting from simplified equations or incomplete physical representations [210–212]. This capability enhances their applicability in practical forecasting scenarios, in which observational data often include complex phenomena beyond the scope of numerical models. Thus, neural operators generalize better to out-of-sample conditions and unforeseen extreme events, improving predictive robustness. Many data-driven forecasting models can be effectively understood within this broader neural operator framework, highlighting their flexibility and efficacy in tackling complex weather- and climate-prediction challenges.

Among neural operators, Fourier neural operators (FNOs) are widely utilized [8]. This technique leverages the powerful representation capabilities of Fourier transforms, capturing spatial correlations across multiple scales. In weather and climate forecasting, FourCastNet utilizes the framework of FNOs to achieve a highly efficient, data-driven approach while leveraging physical insights [192]. FourCastNet uses spherical convolutions or spectral transformations to ensure that predictions respect global properties on Earth’s spherical geometry. WeatherGFT proposes a hybrid physics–AI model that generalizes weather forecasts to finer-grained temporal scales beyond the training dataset [213]. More precisely, they employ a carefully designed PDE kernel to simulate physical evolution on a small timescale (e.g. 300 s) and use a parallel neural network with a learnable router for bias correction. Furthermore, WeatherGFT introduces a lead-time-aware training framework to promote the generalization of the model at different lead times. The weight analysis of physics–AI modules indicates that physics conducts major evolution while AI performs corrections adaptively.

NeuralGCM and ClimODE are further examples of hybrid frameworks that integrate physical principles into DL architectures [13,214]. These models often start with a structure inspired by general circulation equations—describing fluid motion on rotating Earth—and embed trainable components that learn the subgrid-scale processes or parameterizations. By respecting conservation laws and large-scale atmospheric balances, NeuralGCM can focus their learning capacity on complex physical parameterizations (e.g. cloud microphysics, radiation or ocean–atmosphere coupling). Similarly, ClimODE seeks to merge neural ordinary differential equation structures with convective dynamics, ensuring that the physical backbone remains intact. These strategies reduce the risk of unphysical results while harnessing AI for tasks such as parameter estimation, downscaling or DA.

A crucial motivation for these hybrid physics–AI approaches is that purely data-driven methods may fail when encountering conditions beyond their training distribution. Extreme-weather events, long-term climate shifts and complex interactions between land, ocean and atmosphere processes are difficult to capture without explicit physical constraints [41]. By integrating domain knowledge directly into AI architectures, one can reduce overfitting and ensure more robust extrapolation. Furthermore, hybrid physics–AI methods often require fewer labeled data points than purely data-driven approaches, as the physical components guide or regularize the learning process [215]. This synergy can be particularly beneficial in regions or at timescales with sparse observations.

However, hybrid physics–AI models face several challenges. Foremost among them is the complexity of integrating high-fidelity physical equations into trainable architectures without making them computationally unwieldy. Most PIML methods may struggle when the domain is complex or when the underlying dynamics involve significant turbulence or stochasticity [216]. Balancing accuracy and efficiency often requires the careful design of model architectures, solvers and DA schemes. Another issue is interpretability: while physics-based equations are transparent, neural network representations are not. One must ensure that the hybrid designs remain explainable enough to build trust with end users [217].

## AI-MIP (AI-BASED MODEL INTERCOMPARISON PROJECT)

**Figure 8.**
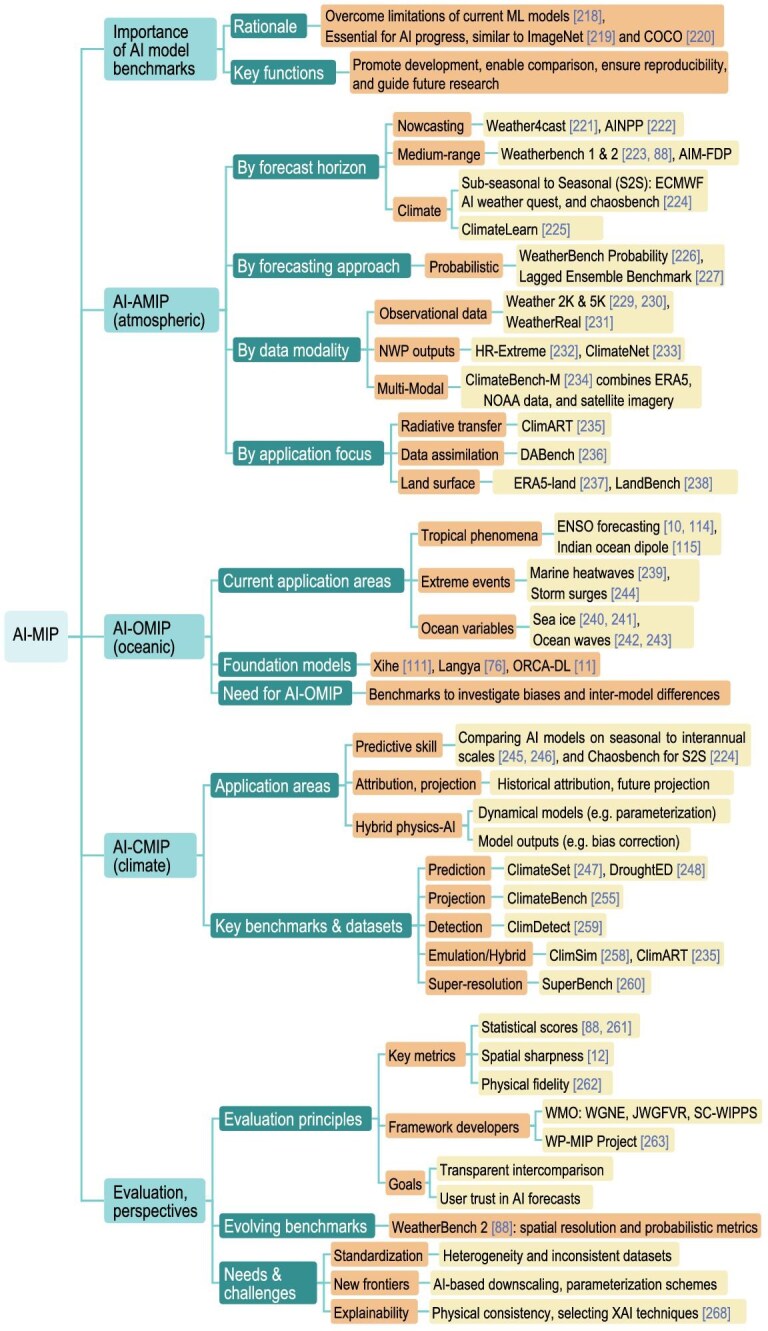
Schematic diagram of the AI-MIP (Model Intercomparison Project).

In recent years, AI-based weather and climate foundation models have demonstrated advanced forecasting capabilities, but with critical challenges remaining unresolved, as discussed above [218]. Standardized benchmarks with high-quality data, such as ImageNet [219] and COCO [220], have driven progress by providing structured datasets, well-defined objectives and rigorous evaluation protocols. Similarly, comprehensive benchmarks are also crucial to the development of weather and climate AI models by (i) broadening participation, (ii) enabling systematic comparison, (iii) ensuring reproducibility, (iv) assessing model applicability, (v) tackling model limitations and (vi) guiding future directions (Fig. 8).

### AI-AMIP (AI-based atmospheric model intercomparison project)

Current benchmarks for AI-based atmosphere models can be classified by forecast horizon, forecasting approach, data modality and application focus.

Regarding forecast horizon, benchmarks cover nowcasting, medium-range, subseasonal and climate-scale forecasting. Weather4cast targets precipitation nowcasting by using satellite data [221], while the AI for Nowcasting Pilot Project (AINPP) of the World Meteorological Organization (WMO) focuses on operational nowcasting [222]. For medium-range (1–15 days’) forecasting, WeatherBench [223] and WeatherBench2 [88] provide standardized datasets and evaluation metrics. The AI Model Forecast Demonstration Project (AIM-FDP) of China Meteorological Administration supports the operational deployment of AI-based medium-range weather-forecasting models. For S2S forecasting, ECMWF’s AI Weather Quest and ChaosBench [224] foster AI-based S2S advancements. ClimateLearn provides an open-source library for climate AI research [225].

Forecasting approaches include deterministic and probabilistic methods. While early benchmarks focused on deterministic metrics, recent benchmarks, such as WeatherBench2, WeatherBench Probability [226] and Lagged Ensemble Benchmark [227], incorporate probabilistic scores such as the continuous ranked probability score, Brier score and spread-skill ratio.

In terms of data modality, benchmarks utilize reanalysis (e.g. ERA5), NWP outputs, satellite observations and *in situ* measurements. Despite its popularity, ERA5 has known biases, particularly in precipitation [228] and tropical cyclone intensity [102]. Benchmarks such as Weather2K [229], WEATHER-5K [230] and WeatherReal [231] integrate observational datasets, while HR-Extreme [232] and ClimateNet [233] include NWP outputs. ClimateBench-M [234] combines ERA5, NOAA extreme-weather-event data and satellite imagery.

Application encompasses general-purpose forecasting and specialized tasks, including extreme-event prediction, radiative transfer (e.g. ClimART [235]), DABench [236]) and land-surface prediction (ERA5-land [237], LandBench [238]).

### AI-OMIP (AI-based ocean model intercomparison project)

AI has also been applied to predict tropical air–sea coupled modes [10,114,115], mesoscale eddies, marine heatwaves [239], sea ice [240,241], ocean waves [242,243] and storm surges [244]. Recent foundation models [11] outperform conventional statistical and dynamical models [239]. The growing diversity of AI-based ocean-forecasting models underscores the need for an AI-ocean-model benchmark, with unified metrics and standardized datasets to systematically assess model performance and biases as well as forecast uncertainties.

### AI-CMIP (AI-Climate Model Intercomparison Project)

The AI-based Climate Model Intercomparison Project (AI-CMIP) can be grouped into predictive-skill assessment, climate attribution/projection and a hybrid physics–AI model.

For predictive-skill assessment on seasonal-to-interannual timescales [245,246], model heterogeneity in architectures, training data and evaluation protocols complicates universal benchmarking. ClimateSet [247] offers large and consistent datasets derived from 36 Input4MIPs and CMIP6 models. Specialized datasets further support applications for drought [248] and extreme-weather events [249].

For attribution and projection, AI offers efficient alternatives to computationally intensive ESMs [250–254]. ClimateBench [255] establishes a unified framework for evaluating both dynamical and AI models, and is applied broadly in climate tasks [256,257]. ClimSim [258] and ClimDetect [259] provide large-scale datasets for detection and attribution.

Hybrid physics–AI models are increasingly applied for physical parameterization, bias correction, downscaling and post-processing. SuperBench [260] provides high-resolution datasets, though comparable climate downscaling datasets remain limited. ClimART [235] offers large-scale benchmark data for atmospheric radiative transfer.

### Guiding principles for evaluation protocol and metrics

In contrast to conventional physics-based models, AI models are trained on data rather than governed by physical equations. This fundamental paradigm shift introduces distinct challenges for model evaluation and intercomparison, particularly in assessing the robustness, generalization, stability, physical consistency, dynamical balance and conservation properties of AI models [261]. Therefore, evaluating AI-based weather and climate foundation models must go beyond standardized benchmarks and include a broader set of verification diagnostics. These should combine traditional statistical skill scores [88,223], measures of spatial sharpness or smoothness [12] and targeted tests of physical consistency and process fidelity [262]. This multidimensional evaluation framework is critical to ensure that AI models are not only skillful, but also physically credible and reliable for scientific and operational use.

To address these needs, WMO expert groups, including the Working Group on Numerical Experimentation (WGNE), the Joint Working Group on Forecast Verification Research (JWGFVR) and the Standing Committee on WMO Integrated Processing and Prediction System (SC-WIPPS), are developing evaluation frameworks through the Weather Prediction Model Intercomparison Project (WP-MIP) [263] to enable fair and systematic comparison of AI-based, physics-based and hybrid forecasting systems, while explicitly accounting for the unique characteristics and limitations of AI models. Establishing such standards is essential for transparent intercomparison with traditional approaches and for building user trust in AI-based forecasts.

### Perspective

As AI-based foundation models advance, benchmarks must evolve accordingly. For instance, WeatherBench2 improves upon its predecessor by enhancing the spatial resolution to 0.25° and incorporating probabilistic metrics, reflecting the superior probabilistic forecasting performance of AI relative to NWP models [13,104,264]. Evaluating AI-based models requires domain-specific expertise [265,266]. To promote broader participation, open-source evaluation platforms such as WeatherBench2 promote broader participation by collecting models for standardized evaluation.

Despite progress, ocean and climate AI models remain highly heterogeneous, complicating unified benchmarking. While ChaosBench and ClimateBench provide important foundations, further efforts are needed to rigorously assess model skill, physical consistency and explainability [267], particularly for downscaling and physical parameterization.

## USEFULNESS AND APPLICABILITY OF AI MODELS TO SOCIETY

AI is emerging as a disruptive technology, profoundly reshaping various societal sectors by offering high-resolution forecasts at significantly lower computational costs [268]. AI-driven systems excel at handling high-dimensional datasets and identifying subtle patterns among weather variables, which enables them to deliver faster and more energy-efficient forecasts. These advancements provide direct benefits to critical sectors. In the energy industry, AI offers precise generation forecasts for wind and solar installations, meeting their unique operational needs [269], and demonstrates superior performance in both accuracy and efficiency [270]. In agriculture, AI assists farmers in optimizing planting schedules and crop selection based on weather outlooks, thereby enhancing productivity and sustainability. Furthermore, leveraging the integration of meteorological and geospatial foundation models through AI can effectively enhance the development of multi-hazard early-warning systems [271]. The global market for AI-based climate modeling is expanding rapidly, projected to grow at a compound annual growth rate of >23% between 2025 and 2034, reflecting increasing investment and adoption by governments and industries seeking data-driven strategies for climate resilience.

However, the widespread application of AI models in atmosphere–ocean sciences faces significant challenges that temper their operational adoption. A primary concern is the reliability of the models in forecasting unprecedented extreme events or ‘gray swans’ [272]. Furthermore, specific applications face data limitations. For instance, many radar-data-based AI nowcasting systems cannot provide a comprehensive, high-resolution view of all relevant factors. Ensuring equitable access and governance also remains a critical challenge, as gaps in digital infrastructure and the presence of algorithmic bias can limit the effectiveness of these models, particularly in the southern hemisphere [273].

To fully unlock the societal potential of AI, future development must address these challenges. A critical step is the systematic comparison of traditional NWP and AI models to better understand how their fundamental differences in efficiency and methodology impact forecast outcomes [272,274]. Moreover, it is crucial to move beyond statistical accuracy and quantify the tangible value that AI improvements provide for end users. A more statistically accurate forecast does not automatically translate into a better decision [272], highlighting the need to co-design and evaluate AI solutions with stakeholders in sectors such as energy, transportation and agriculture to measure their real-world impact. This involves developing robust, accountable and fair AI pipelines that support evidence-based policymaking and operational use. Despite these hurdles, the market trend shows a clear and growing integration of AI into operational weather services worldwide, signaling that their immense potential is recognized. This trajectory points toward a new era of intelligent forecasting, in which data-driven insights and physical laws are deeply intertwined to build a more resilient and sustainable society [269].

## AI AGENTS FOR CLIMATE SCIENCE (AS ONE GROUND-BREAKING WAY FORWARD)

AI is transforming science, not only by accelerating simulations and predictions, but also by acting as a genuine partner in discovery. In materials science, autonomous laboratories guided by intelligent agents are discovering new compounds and catalysts [275]. In life sciences, general-purpose biomedical agents and virtual laboratories of AI systems are designing experiments, proposing hypotheses and even discovering novel therapeutics such as nanobodies [276,277]. These advances highlight a broader trend toward the emergence of the ‘AI scientist’, in which machines evolve from analytical tools into collaborative partners that support and extend human intellect.

**Figure 9.**
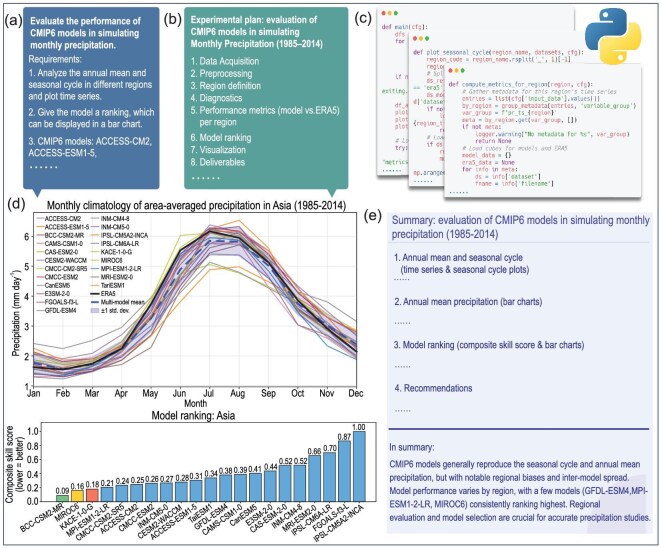
Evaluation of CMIP6 models in simulating seasonal cycles of monthly precipitation in Asia by EarthLink. (a) Task definition and diagnostic requirements, including annual mean and seasonal cycles and regional time-series analyses. (b) Automated planning output from EarthLink, detailing end-to-end workflow from data acquisition to deliverables. (c) Example code snippet generated by the system for data processing and analysis. (d) Modeled and observed precipitation seasonal cycles over the selected region, with annual mean ranking and model performance comparison. (e) Automated textual interpretation of the results, providing a plain-language summary generated by the system.

Earth science is beginning to follow this trajectory. The central challenge in this domain is not simply to improve prediction from ever larger datasets, but to achieve a deeper, human-like understanding of the physical world, especially when working with sparse and heterogeneous observations. Meeting this challenge requires moving beyond pattern recognition toward genuine scientific reasoning. A step in this direction is represented by EarthLink—an AI agent system designed as an interactive copilot for climate scientists [278]. Researchers can pose questions in natural language and EarthLink interprets intent, generates experimental plans, writes analysis code and synthesizes results into transparent and traceable research reports, such as evaluating the annual precipitation cycle in Asia (Fig. 9). By learning from successful cases through a practice–learning–improvement cycle, EarthLink continuously evolves to address increasingly complex problems. Rather than replacing researchers, it elevates their role from data processors to strategic directors of inquiry, enabling them to focus on hypothesis generation and creative exploration.

Looking ahead, the most powerful advances will come from integrating predictive models with reasoning agents. State-of-the-art forecasting systems, which already achieve remarkable accuracy, can be coupled with agents such as EarthLink to form a forecast-to-action framework in which AI not only predicts events, but also investigates their underlying drivers, evaluates potential impacts and proposes mitigation strategies. Such a synthesis would represent a profound step toward truly collaborative science, allowing humans and machines to jointly confront the complex challenges of a changing climate.
